# Traditional Chinese Medicine Zheng in the Era of Evidence-Based Medicine: A Literature Analysis

**DOI:** 10.1155/2012/409568

**Published:** 2012-06-06

**Authors:** Miao Jiang, Chi Zhang, Guang Zheng, Hongtao Guo, Li Li, Jing Yang, Cheng Lu, Wei Jia, Aiping Lu

**Affiliations:** ^1^Institute of Basic Research in Clinical Medicine, China Academy of Chinese Medical Science, Beijing 100700, China; ^2^School of Information Science and Engineering, Lanzhou University, Lanzhou 730000, China; ^3^Department of Nutrition, University of North Carolina at Greensboro, North Carolina Research Campus, Kannapolis, NC 28081, USA; ^4^School of Chinese Medicine, Hong Kong Baptist University, Kowloon Tong, Kowloon, Hong Kong; ^5^E-Institute of Shanghai Municipal Education Commission, Shanghai TCM University, Shanghai 201203, China

## Abstract

Zheng, which is also called a syndrome or pattern, is the basic unit and a key concept of traditional Chinese medicine (TCM) theory. Zheng can be considered a further stratification of patients when it is integrated with biomedical diagnoses in clinical practice to achieve higher efficacies. In an era of evidence-based medicine, confronted with the vast and increasing volume of TCM data, there is an urgent need to explore these resources effectively using techniques of knowledge discovery in databases. The application of effective data mining in the analysis of multiple extensively integrated databases can supply new information about TCM Zheng research. In this paper, we screened the published literature on TCM Zheng-related studies in the SinoMed and PubMed databases with a novel data mining approach to obtain an overview of the Zheng research landscape in the hope of contributing to a better understanding of TCM Zheng in the era of evidence-based medicine. In our results, contrast was found in Zheng in different studies, and several determinants of Zheng were identified. The data described in this paper can be used to assess Zheng research studies based on the title and certain characteristics of the abstract. These findings will benefit modern TCM Zheng-related studies and guide future Zheng study efforts.

## 1. Introduction

In traditional Chinese medicine (TCM) theory, Zheng, which is also called a syndrome or pattern, is the basic unit and a key concept. TCM Zheng is the abstraction of a major disharmonious pathogenesis, which is identified from a comprehensive analysis of clinical information from four main diagnostic TCM methods: observation, listening, questioning, and pulse analyses [1]. In brief, all diagnostic and therapeutic methods in TCM are based on the differentiation of TCM Zheng, a concept that has been used in China for over 3,000 years [2, 3].

TCM Zheng can be understood as a guideline for patient classification in clinical practice from an alternate viewpoint/dimension compared to a biomedical disease diagnosis. For example, patients suffering from the same disease might be classified with different TCM Zhengs, whereas different diseases might be categorized with the same TCM Zheng. Different Zhengs may occur for one patient at the same time, and Zheng classification is dynamic because Zheng can change during the evolution of a disease. Thus, TCM Zheng classification could be considered to be a further stratification in patients with a single disease, allowing clinicians to obtain more accurate patient classifications. At present, a TCM Zheng diagnosis is integrated with a biomedical diagnosis in clinical practice, and integrative medicine emerges as an optimal approach for achieving higher efficacy [1].

However, in the era of evidence-based medicine, TCM Zheng has encountered a strong challenge from biomedical science due to a shortage of evidence-based theoretical interpretations and solid proof of Zheng-based efficacy. Therefore, researchers have made a great deal of effort in TCM Zheng-related studies and have made considerable achievements in this field. For instance, it has been indicated that TCM Zheng classification based on symptoms can be used for further stratification of patients with rheumatoid arthritis, which can improve the efficacy of the selected biomedical intervention [4]. In addition, TCM Zheng classification would help to build up a molecular network of TCM Zheng classification in certain diseases, which would help to decipher the mechanism of TCM Zheng classification and define the potential mechanisms of herbal medicines [5, 6]. In recent years, TCM Zheng has attracted increasing attention; it has been shown that this specific patient classification method could assist in new findings for medical science if it were adopted as a significant diagnostic method in modern TCM research with regard to diagnoses, clinical trials, and new drug discoveries [7].

In the past two decades, studies in TCM Zheng have increased dramatically along with advances in medical technologies. Confronted with the large and increasing volume of TCM data, an urgent need emerges to explore these resources effectively using techniques of knowledge discovery in databases (KDD) [8]. We believe that effective data mining approach applications in the analysis of multiple extensively integrated databases (such as the TCM database SinoMed for TCM Zheng classification and the PubMed database for biomedicine) can supply new information in TCM Zheng research, including findings regarding the basic rules of Zheng distribution in certain diseases; the correlations between Zheng, disease, and herbal prescriptions; and the build-up of Zheng-Zheng and Zheng-disease correlation networks. These findings will benefit modern TCM Zheng-related studies.

In this study, we screened the published literature on TCM Zheng-related studies in the SinoMed and PubMed databases with a novel data mining approach to review the Zheng research landscape with the hope of contributing to a better understanding of TCM Zheng in the era of evidence-based medicine.

## 2. Materials and Methods

### 2.1. Materials: Source Data Collection

The majority TCM studies were found in the Chinese-language database SinoMed. Most modern TCM research studies were found in the English-language database PubMed. Thus, the TCM Zheng database was separated into two groups. The relevant studies were downloaded from PubMed (http://www.ncbi.nlm.nih.gov/PubMed/) and SinoMed (http://sinomed.imicams.ac.cn/zh/b/index.jsp).

#### 2.1.1. Chinese Literature from the SinoMed Database

By querying the term “*中医证候*” (TCM Zheng) within the scope of title, keyword, and abstract, SinoMed returned a dataset containing 275,408 articles on December 11, 2011. In the procedure of data preparation, we found that there are much fewer publications (less than 5% of total records) before the year 1990, with questionable study quality comparing with recent publications, thus the data before 1990 were ignored in this study which included 11,378 records. Therefore, the dataset after 1990 contains 266,160 records.

#### 2.1.2. English Literature from the PubMed Database

By querying the term “TCM Zheng/syndrome/pattern” on the default query search, PubMed returned a dataset of 28,103 articles on December 11, 2011.

### 2.2. Methods: Data Processing

In this study, TCM Zheng can be classified by several grouping policies. For each group policy, different statistical methods that were based on similar algorithms were adapted.

Because there was a delay in the literature collection process, the 2011 dataset was not completed until December 11, 2011. Therefore, not all of the data that were tagged with the year 2011 were included in all of the annual statistics in this study.

#### 2.2.1. TCM Zheng Studies in the Chinese Literature

First, according to the carriers of TCM Zheng studies, the studies can be classified into three groups. Group one includes all TCM Zheng animal experimental studies. Group two includes all TCM Zheng clinical studies, and group three includes TCM Zheng theoretical studies and involved neither animal model nor clinical studies.

**Figure 1:**
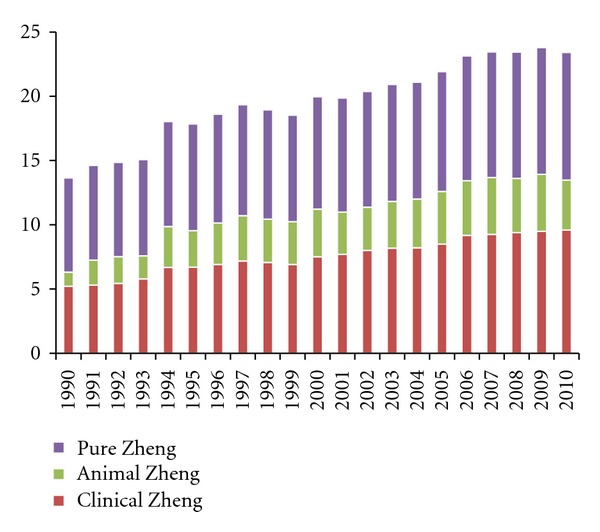
Annual distribution of Chinese-language articles about TCM Zheng in 3 categories (animal experimental studies, clinical studies, and pure Zheng-related studies). The data are obtained from the SinoMed database (until December 11, 2011). In the calculation, some of the annual frequencies of animal studies are 0 and 1, which are too small to be clearly shown in the column diagram. Therefore, the values are converted by the natural logarithm function “Annual Value = ln(Annual Value_origin_ + 1).” Based on this function, the frequency of 0 is still 0, and the frequency of 1 (as well as other values) is ln(2).

According to these three groups, the TCM Zheng statistics were focused on animal studies, clinical research, and pure TCM Zheng studies (nonanimal or nonclinical). The result is shown in Figure 1.

#### 2.2.2. Studies of TCM Zheng and Diseases in Chinese Literature

Many studies on TCM Zheng involved biomedical diseases, so the statistics included studies that involved diseases and those that did not involve diseases.

**Figure 2:**
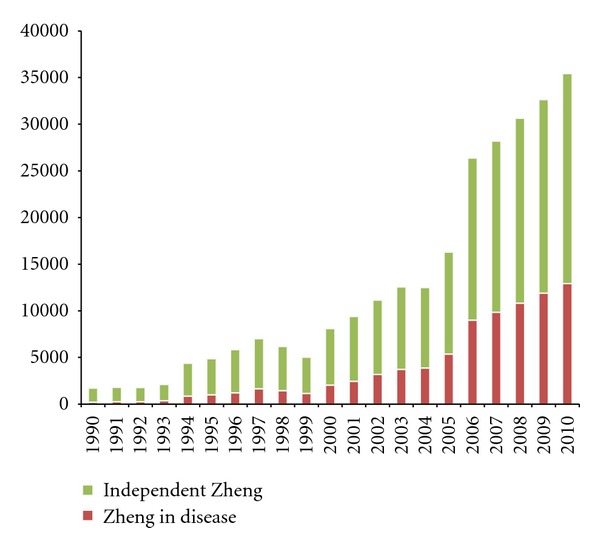
The annual records of Chinese-language articles about TCM Zheng classification in 2 categories. The data were obtained from the SinoMed database (until December 11, 2011). The statistics are based on scanning studies as to whether they contain a disease name in the framework of Western medicine. The count of “independent Zheng” increases by one if a study does not contain a disease name, and the count of “Zheng in disease” increases by one if the study contains one or more disease names.

In analyzing the Chinese literature, we filtered by title, keyword, and abstract. The count of independent Zheng studies increases by one if no disease name occurred; otherwise, the count of disease-related Zheng increases by one. The result was shown in Figure 2.

#### 2.2.3. TCM Zheng Studies in the English Literature

Similar to Section 2.2.1, TCM Zheng studies from the PubMed database were grouped into three classes: pure Zheng studies, clinical Zheng studies, and animal Zheng studies.

**Figure 3:**
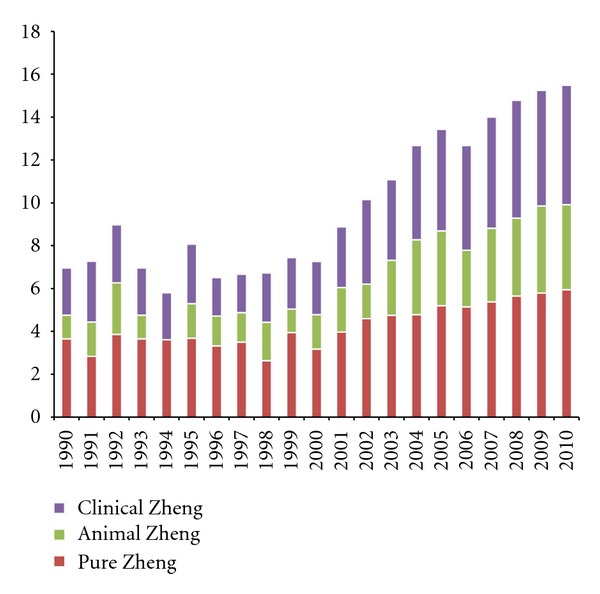
The annual records of English-language articles about TCM Zheng classification in 3 categories. The data were obtained from the PubMed database (until December 11, 2011). As in Figure 1, the annual values are also converted by the function “Annual Value = ln(Annual Value_origin_ + 1).” Therefore, a comparison between SinoMed and PubMed can be obtained based on the same standard.

The statistical method was similar to that of Section 2.2.1. The only difference was that the two methods were focused on different languages (Chinese versus English, resp.), as shown in Figure 3.

#### 2.2.4. The Ten Most Common Diseases Associated with Chinese Zheng Studies

By limiting the TCM Zheng literature to clinical studies, we obtained the frequencies of studies related to different diseases. The 10 most commonly associated diseases were listed in Table 1.

#### 2.2.5. Annual Distribution of 10 Most Common Diseases Associated with TCM Zheng

**Figure 4:**
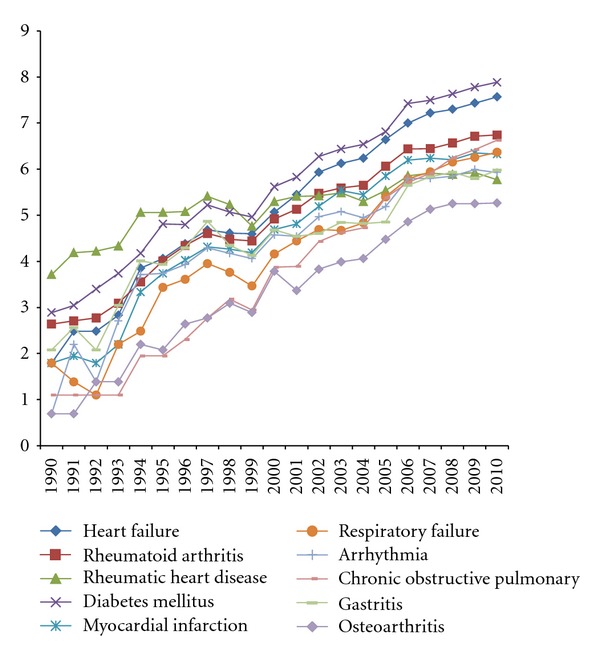
The ten most common diseases in Chinese-language TCM Zheng-related clinical studies annually. The data were obtained from the SinoMed database (until December 11, 2011). Each line represents the annual studies of TCM Zheng for one particular disease. The annual values are converted by the function “Annual Value = ln(Annual Value_origin_ + 1)” to better display the tendency.

According to the results of Section 2.2.4 and Table 1, we filtered the Chinese studies that were associated with these 10 diseases and separate them into 10 datasets. By analyzing these datasets with respect to their dates of publication, we obtained their annual distributions, shown in Figure 4.

#### 2.2.6. Zheng-Zheng Network Generated from the Chinese Literature

**Figure 5:**
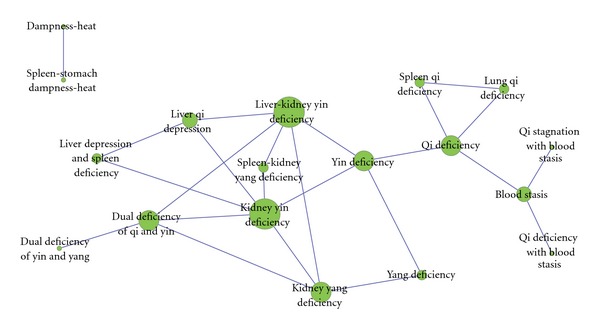
Overview of the Zheng-Zheng network. This network is generated from mining the SinoMed literature on TCM Zheng. The method of calculation is based on a data slicing algorithm that calculates the frequencies of the co-occurrence TCM Zhengs. Each node represents one type of Zheng. The size of the node indicates the frequency of Zheng publications; a larger node indicates more reports about the Zheng. The line width represents the frequency of co-occurrence of the connected Zhengs.

Based on the cooccurrence of TCM Zheng and applying the data slicing algorithm [9], we obtained the Zheng-Zheng network, shown in Figure 5.

#### 2.2.7. The Twenty Most Common Zhengs and Their Associated Diseases

Because there was a strong connection between TCM Zheng and disease in both clinical practice and research studies, it was necessary to obtain the frequencies of different disease-Zheng association items that commonly existed in the Chinese literature.

By analyzing the literature associated with both TCM Zheng and disease names in a framework of Western medicine, we obtained a list of associated items of disease-Zheng and their frequencies. For simplicity, we list the 20 most common in Table 2.

#### 2.2.8. Disease-Zheng Network Generated from the Chinese Literature

Because one disease could be involved with several Zhengs, it is necessary to explore the major Zhengs that are associated with each particular disease.

**Figure 6:**
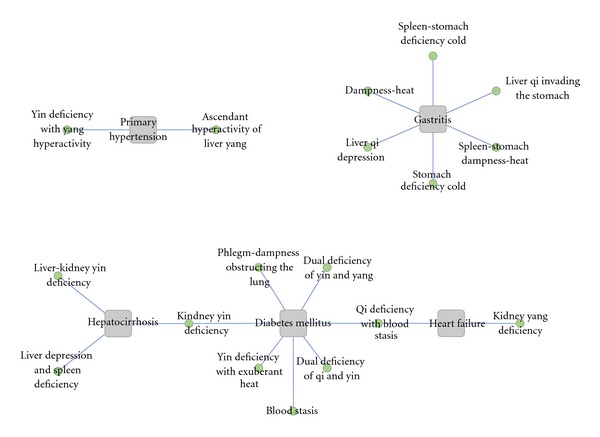
Overview of the disease-Zheng network. The disease-Zheng network is generated from a SinoMed literature analysis with the cooccurrence frequencies of disease. The method of calculating the frequency of co-occurrence is also based on a data slicing algorithm. In this figure, the square grey shape is a certain disease, and the round green shape is a TCM Zheng. If two diseases have a common Zheng, there is an edge connecting them. The upper left part identifies the relevant Zheng research on primary hypertension, with 2 TCM Zhengs for this disease. The upper right part represents the 6 most influential Zhengs in gastritis research. The section below each part represents the total number of shared Zheng among diabetes mellitus, hepatocirrhosis, and heart failure. The kidney yin deficiency Zheng can be found in both DM and Hepatocirrhosis, and Qi deficiency with blood stasis Zheng can be found in both DM and HF.

These statistics were focused on the cooccurrence of disease names and Zheng terms. By analyzing the Chinese literature, we obtained a disease-Zheng network. In Figure 6, we listed the 5 most common diseases and their associated Zhengs.

## 3. Results

In total, 266,160 Chinese-language studies on TCM Zheng were obtained from the SinoMed database, and 28,103 English-language studies were obtained from PubMed. All analyses were performed based on these studies.

### 3.1. Overall Literature Profiles of Zheng-Related Research

Figure 1 showed an annual increase in the number of publications in the SinoMed database. The number of articles has increased rapidly in the past 2 decades. In addition, the portion of clinical studies has increased substantially, especially after 2006. Animal experimental studies remained insignificant, and the numbers of related articles remained a small proportion of the total, indicating that animal experimentation has not been a major part of Zheng-related studies.

As a diagnostic method, TCM Zheng diagnosis can be integrated with a biomedical diagnosis in clinical practice, thus we can classify the whole studies into two categories, independent Zheng and Zheng in disease. The former indicates those studies considering only TCM Zheng classification without any biomedical disease information; the Zheng in disease studies refers to those studies aiming at the TCM Zheng research based on one or more biomedical diseases, or the integrative study on TCM Zheng and biomedical diseases. The majority of studies are independent of biomedical disease, as shown in Figure 2, confirming that TCM Zheng classification can be discussed as a different classification system independent of disease diagnosis, although the integration of Zheng and disease diagnosis is common in clinical practice. The proportion of studies that were correlated with biomedical diseases is increasing over time, especially after the year 2000. The advantage of integrating TCM Zheng with biomedical disease diagnoses has been emphasized in recent years, and a number of novel achievements have been acquired in this field.

After 2000, the annual number of articles in English-language journals on TCM Zheng in PubMed increased dramatically, but the total number was far less than the number of Chinese-language articles, as shown in Figure 3. Among these studies, the percentage of clinical studies grew rapidly, a trend that was consistent with that of Chinese-language studies. A higher proportion of animal experimental studies was reported in PubMed than in SinoMed.

The 10 most common diseases in Chinese-language TCM Zheng-related studies are summarized in Table 1, and the annual numbers are shown in Figure 4. From Table 1 and Figure 4, it can be concluded that most of the TCM Zheng-related diseases are complex chronic diseases, which implies that researchers tend to focus on these chronic diseases in TCM Zheng-related studies due to the superior efficacy of herbal prescriptions in treating these diseases. There are thousands of studies per year focusing on TCM Zheng studies of diabetes mellitus and gastritis, and both of these diseases manifest with multiple symptoms with an increasing incidence in China and can be treated with herbal medicines.

### 3.2. Basic Zhengs and the Zheng-Zheng Association Analysis

As a basic unit in a TCM diagnosis, Zheng can be shown in combination (two or more Zhengs) in a patient, and Zheng can change during the development of an illness. During the data analysis, it can be found that most disease-Zhengs studies are published in Chinese. Although there are a small amount of English publications concerning the disease-Zheng research, most of them were published in English abstract, which actually were published in Chinese, and can be collected in SinoMed database. Thus we abandoned the English data in this analysis, for the data is too few, and also it is not appropriate in this study to combine both data together. There are 18 basic Zhengs that are filtered out in the TCM publications in SinoMed. Figure 5 illustrates those Zhengs and the Zheng-Zheng association network. Clockwise from the largest node, the first is the liver-kidney yin deficiency pattern (connecting five nodes: yin deficiency pattern, kidney yang deficiency pattern, spleen-kidney yang deficiency pattern, pattern of dual deficiency of qi and yin, and liver qi depression pattern). Six nodes of the network are connected to the second largest node kidney yin deficiency pattern. The yang deficiency pattern and pattern of dual deficiency of yin and yang are two patterns with relatively low frequencies. The upper left corner is the dampness-heat pattern and connecting node spleen-stomach dampness-heat pattern. The upper right is the qi deficiency pattern, connecting with the spleen qi deficiency pattern and lung qi deficiency pattern. The lower right is the blood stasis pattern, connecting with the pattern of qi deficiency with blood stasis and pattern of qi stagnation with blood stasis.

### 3.3. Disease-Zheng Association Analysis

The integration of disease diagnosis and TCM Zheng classification is a common model in clinical practice, and many studies have focused on this integration. According to Zheng-Zheng association analysis in Section 3.2, most disease-Zhengs studies are published in Chinese, and English data were abandoned for the small quantity; we then developed an approach to visualization that classifies data according to disease-zheng association analysis. Details of the top 20 frequent disease-Zheng (Zheng in a specific disease) are provided in Table 2. In the pattern distribution, the patterns with yin deficiency were the most frequent (1,794; 44.89%), and the two TCM viscera (liver and kidney, internal organs where essence and qi are formed and stored in TCM) were the most frequent (1,151; 28.80%).

To further confirm the disease-Zheng associations, 20 disease-Zheng were selected for more comprehensive analyses. Figure 6 reveals insights into the disease-Zheng association; it was built by analyzing 5 kinds of popular diseases. The constructed view shows three attributes. The first (upper left) attributes identify the relevant Zheng research on primary hypertension (PH); there are 2 TCM Zheng for PH. The second (upper right) attributes represent the 6 most influential Zheng in gastritis research. The third attribute represents the total number of shared Zheng among diabetes mellitus (DM), hepatocirrhosis, and HF. Kidney yin deficiency Zheng can be found in both DM and Hepatocirrhosis, and Qi deficiency with blood stasis Zheng can be found in both DM and HF.

## 4. Discussion

Compared to a previous literature review [10, 11], we report a new quantitative route for the synthesis of related literature and provide new quantitative evidence on TCM Zheng studies.

A central problem is how to capture information from literature in a form that is suitable for analysis [12]. We address the information on Zheng and show that the frequencies of words in abstracts can be used to determine whether or not a given article discusses Zheng. For those articles that have been determined to discuss this topic, relevant information can be obtained. Furthermore, suitable annotations can be obtained. These evaluations are based on limited but increasing evidence from animal studies and clinical studies. Among other limitations, the lack of quantitative assessment has consistently been cited as a fundamental problem in existing studies, and mining exploration has been used in a recent review [1]. The purpose of this study was to provide a comprehensive overview of quantitative levels.

Over the past 30 years, an increasing number of Chinese researchers have focused their attention on developing evidence for Zheng and identifying the mechanism of Zheng. Recently, more studies were published in SCI indexed journals to introduce and evaluate the effectiveness of Zheng.

For Zheng, the highest numbers of Chinese-language articles were reported for experts' experiences, reviews, commentaries, animal studies, observational studies, and randomized controlled trials (RCTs). However, relatively low numbers were reported for animal studies and RCTs. It is difficult to develop an animal model that perfectly reproduces the symptoms of Zheng in patients [13]. Researchers attempt to overcome this limitation by combining the disease and Zheng [14]. The result shows the unambiguous identification of the authors' characteristics. Chinese authors are becoming more aggressive about submitting animal experimental studies for Zheng. However, it is important to note that many Chinese scientists in international institutes bring innovation to worldwide TCM Zheng research. We believe that there is a growing trend of collaboration in combining a disease and Zheng between TCM researchers and biomedical scientists in animal experimental Zheng studies. RCTs were not developed until the 1990s. Recently, more advanced trial designs are being developed and will provide explicit Zheng theories based on long-term experience [15, 16]. Nonetheless, there is a relatively small amount of evidence regarding RCTs with disease and Zheng designs for data mining.

The yin deficiency pattern is currently the preferred pattern for Zheng research compared to any other pattern because it is relatively major component of modern life. A yin deficiency may be due to excessive fluid loss or to the consumption of yin due to aging. As stated in the Nei Jing (Inner Classic), “At 40 years of age, yin is half consumed” [17]. If, due to overthinking, anxiety and worry, underexercise, faulty diet or erroneous medical treatments in modern life, the qi is damaged and becomes vacuous and weak, then the spleen will not be able to perform its various functions. As mentioned above, if yin does not nourish and enrich the liver and kidney, then the liver and kidney will not be able to governing coursing and discharging. Hence, the liver and kidney will become depressed. Thus, it is clear that liver and kidney deficiencies are mutually engendering in the mining results. For the yin deficiency pattern, more research is needed to investigate its contribution to preventing and reversing chronic diseases that are consequences of a modern lifestyle.

Similarly, damp heat typically complicates the diseases of many patients. In addition, dampness can be engendered internally, often due to spicy foods, alcohol, sugars, and sweets. Blood stasis is also a mechanism that is involved in most chronic disorders, especially when there is chronic severe pain at fixed locations. A study on blood stasis and activating blood circulation and removing stasis won the top prize of the National Science & Technology Progress Award in China [18]. In addition, there is less information available on the yang deficiency pattern compared to the yin deficiency pattern.

For disease and Zheng correlation research, the results of all selected studies showed that the number of DM studies was the highest, followed by the number of studies on gastritis and HF. The 5 most common diseases in the mining results are chronic diseases. These chronic diseases are a likely explanation for the report that the yin deficiency pattern is substantially higher than the yang deficiency pattern in Zheng studies, and CM is able to provide a worldwide contribution for patients who suffer from chronic diseases [19].

Similar to DM, more detailed patterns of gastritis were generally consistent with patterns found in clinical practice. However, relatively few mean concentrations for some of patterns were reported for primary hypertension, cirrhosis, and HF.

The results of this study suggest that DM and two diseases, cirrhosis and HF, share one common Zheng. One important concept in TCM is “Treating Different Diseases with the Same Therapy” (TDDST), which can be explained as the similar treatment of different diseases that have similar TCM patterns [9]. For disease and Zheng correlation research such as TDDST, explorations of the existing biomedical networks between diseases are challenging.

Despite the notable accomplishments of Zheng in TCM, it is impossible to exaggerate the importance of Zheng classification. We have been able to identify many of the classical formulas with one-to-one relationships to some diseases in the text-mining process. The formulas are often called effective formulas. These disease-TCM formulas are possible future trends in TCM basic and applied research.

In addition, Zheng studies can vary widely depending on actual academic environments. Thus, the availability of a comprehensive database that include Zheng determinants is likely to result in a more accurate and consistent assessment than when the assessment is based only on expert judgments.

There are some limitations to this approach. Because Zheng is a complex concept, many studies were selected, which complicated comparisons across studies that focus on different research methods. In addition, the classification of Zheng varies with conditions and the “standard” application of Zheng, which have changed over time [20, 21]. Additionally, experimental study records, clinical study records and other relevant records of Zheng were selected to investigate time trends. The proportion of nonexperimental or clinical studies has decreased, but the proportion of clinical studies has increased annually. However, there were insufficient data available to assess the effect of these changes. Consequently, the incorporation of time trends in review assessments is required to improve the mining method. A further limitation of using published literature is the extraction and interpretation of Zheng from reports that were written by different authors for different purposes. The description of the detailed experimental conditions was often unclear or absent, especially in Chinese-language articles. In addition, published reports may have been biased toward worst-case scenarios. Finally, Zheng in other research fields, such as epidemiological studies, has rarely been reported [22, 23].

## 5. Conclusions and Perspectives

### 5.1. Conclusions

Using this novel text-mining approach, contrast in Zheng was found when comparing different studies, and several determinants of Zheng were identified. The data described in this study can be used to assess Zheng research classifications based on titles and certain characteristics of abstracts. Furthermore, these data can guide efforts for future Zheng studies.

### 5.2. Perspectives

Based on our analysis of the literature, it seems that TCM Zheng-related studies will attract increasing interest worldwide, and more TCM Zheng studies will occur in the near future. In the era of evidence-based medicine, scientists will concentrate on studies that can provide solid evidence for compelling Zheng research, including RCTs, animal experimental studies, and bioinformatics research based on data from human samples instead of pure theoretical debates. Integrative studies on TCM Zheng and biomedical diseases will be a focus because TCM Zheng is considered a powerful tool for patient stratification that can supplement the present classification system based on biomedical disease. Optimal and innovative study designs, especially in Zheng-related clinical research and animal experimental studies, are urgently needed. High-quality, evidence-based studies in TCM Zheng-related research is expected to lead to innovation and breakthrough discoveries to establish a more accurate diagnostic system that will contribute to healthcare systems worldwide.

## Figures and Tables

**Table 1:** Ten most common diseases in Chinese-language TCM Zheng-related clinical studies.

No.	Disease	Frequency
1	Heart failure	7,953
2	Rheumatoid arthritis	5,754
3	Rheumatic heart disease	4,802
4	Diabetes mellitus	4,386
5	Myocardial infarction	3,519
6	Respiratory failure	2,439
7	Arrhythmia	2,021
8	Chronic obstructive lung disease	2,009
9	Gastritis	1,414
10	Osteoarthritis	1,205

**Table 2:** The top 20 most frequent Disease-Zheng in published studies.

No.	Disease	Zheng	Frequency
1	Diabetes mellitus	Dual deficiency of qi and yin	783
2	Diabetes mellitus	Dual deficiency of yin and yang	247
3	Diabetes mellitus	Blood stasis	237
4	Diabetes mellitus	Phlegm- dampness obstructing the lung	184
5	Diabetes mellitus	Kidney yin deficiency	150
6	Diabetes mellitus	Yin deficiency with exuberant heat	128
7	Diabetes mellitus	Qi deficiency with blood stasis	109
8	Gastritis	Liver qi invading the stomach	286
9	Gastritis	Spleen-stomach dampness-heat	250
10	Gastritis	Spleen-stomach deficiency cold	148
11	Gastritis	Stomach deficiency cold	148
12	Gastritis	Dampness-heat	132
13	Gastritis	Liver qi depression	106
14	Heart failure	Qi deficiency with blood stasis	133
15	Heart failure	Kidney yang deficiency	132
16	Hepatocirrhosis	Liver-kidney yin deficiency	126
17	Hepatocirrhosis	Kidney yin deficiency	126
18	Primary hypertension	Ascendant hyperactivity of liver yang	115
19	Primary hypertension	Yin deficiency with yang hyperactivity	115
20	Hepatocirrhosis	Liver depression and spleen deficiency	110
